# Evaluation of histological variants of upper tract urothelial carcinoma as prognostic factor after radical nephroureterectomy

**DOI:** 10.1007/s00345-024-04878-6

**Published:** 2024-04-09

**Authors:** Byeongdo Song, Jung Kwon Kim, Hakmin Lee, Sangchul Lee, Sung Kyu Hong, Seok-Soo Byun, Jong Jin Oh

**Affiliations:** 1 Department of Urology, Hanyang University Guri Hospital, Guri, Kyunggi-Do South Korea 11923; 2 Department of Urology, Seoul National University Bundang Hospital, 300, Gumi-Dong, Bundang-Gu, Seongnam, Kyunggi-Do South Korea 13605; 3 Department of Urology, Seoul National University College of Medicine, Seoul, South Korea

**Keywords:** Urothelial carcinoma, Robot-assisted radical nephroureterectomy, Variant histology, Prognosis

## Abstract

**Purpose:**

To evaluate the impact of variant histology on patients with upper tract urothelial carcinoma (UTUC) survival outcomes.

**Materials and methods:**

A total of 519 patients underwent radical nephroureterectomy without neoadjuvant therapy for UTUC at a single institution between May 2003 and December 2019. Multivariate Cox regression analysis evaluated the impact of variant histology on progression-free survival (PFS), cancer-specific survival (CSS), and overall survival (OS).

**Results:**

Among 84 patients (16.2%) with variant histology, the most frequent variant type was squamous cell differentiation (64.3%), followed by glandular differentiation (25.0%) and sarcomatoid variant (2.4%). They showed pathologically advanced T stage (for ≥ T3, 59.5% vs 33.3%, *p* < 0.001), higher tumor grade (96.4% vs 85.7%, *p* = 0.025), and higher rates of lymph node metastasis (17.9% vs 7.8%, *p* = 0.015), angiolymphatic invasion (41.7% vs 25.7%, *p* = 0.003), tumor necrosis (57.1% vs 29.0%, *p* < 0.001) and positive surgical margin (13.1% vs 5.7%, *p* = 0.015). On multivariate Cox regression analyses, variant histology was significantly associated with worse PFS (hazard ratio [HR] 2.23; 95% confidence interval [CI] 1.55–3.21; *p* < 0.001), CSS (HR 2.67; 95% CI 1.35–5.30; *p* = 0.005) and OS (HR 2.22; 95% CI 1.27–3.88; *p* = 0.005). In subgroup analysis, no significant survival gains of adjuvant chemotherapy occurred in patients with variant histology.

**Conclusions:**

Variant histology was associated with adverse pathologic features and poor survival outcomes. Our results suggest that patients with variant histology may require a close follow-up schedule and novel adjuvant therapy other than chemotherapy postoperatively.

**Supplementary Information:**

The online version contains supplementary material available at 10.1007/s00345-024-04878-6.

## Introduction

Upper urinary tract urothelial carcinoma (UTUC) is a relatively rare genitourinary malignancy, occupying 5–10% of all urothelial carcinoma [1, 2] while showing poor survival outcomes. Approximately one-third of patients with localized UTUC are reported to experience disease recurrence and cancer-related mortality [3, 4] after radical nephroureterectomy (RNU) with ipsilateral bladder cuff excision, which is the gold standard treatment option for high-risk localized UTUC [5]. Therefore, establishing potential or significant prognostic factors of UTUC is necessary for evidence-based decision-making or patient counseling. However, considering the rarity of UTUC, the potential prognostic factors might be extrapolated from those of bladder cancer.

In general, variant histology has been recognized as an adverse risk factor in patients with urothelial carcinoma of the bladder, achieving a consensus that variant histology presents a poor prognosis, especially in non-muscle invasive T1 bladder cancer [6]. Therefore, it could be reasonable to suggest that variant histology might be associated with worse survival outcomes in patients with UTUC, which is histologically like bladder cancer [7].

However, the clinical importance of variant histology in patients with UTUC has not been well established compared with those with bladder cancer, partially due to the rarity of UTUC [1]. The previous studies showed controversial results on the impact of concurrent variant histology on the prognosis of UTUC [8–12]. Therefore, our study aims to evaluate the effect of variant histology on survival outcomes of UTUC after RNU.

## Material and methods

### Patients

A total of 519 patients who underwent RNU for UTUC between May 2003 and December 2019 were enrolled in our retrospective single-institution study. Exclusion criteria were patients with a history of muscle-invasive bladder cancer (MIBC), neoadjuvant therapy, distant metastasis before surgery, or incomplete medical records. In the final cohort, there were no patients with history of dialysis, solitary kidney, or conservative treatment for UTUC before RNU. The surgical method (open, laparoscopic, or robotic) was decided based on each patient’s and surgeon’s preference. Regional lymphadenectomy was performed in patients with suspicious lesions identified in preoperative imaging or those with enlarged lymph nodes intraoperative examination, while the surgeon’s discretion determined the extent of lymphadenectomy.

### Clinicopathologic data and survival outcomes

All surgical specimens after RNU were assessed in detail by a single genitourinary pathologist according to standard pathologic procedures. Pathologic stage and grade for UTUC were evaluated according to the 2002 American Joint Committee on Cancer (AJCC)/Union Internationale Contre le Cancer (UICC) TNM classification and 1998 World Health Organization (WHO)/International Society of Urologic Pathology (ISUP) consensus classification, respectively, as previously reported [13]. Variant histology was evaluated according to the standardized diagnostic criteria described in the WHO classification of tumors [14]. Clinicopathologic data included the patient’s age at RNU, gender, body mass index (BMI), co-morbidity, smoking status, Eastern Cooperative Oncology Group (ECOG) performance status scale, the previous history of gross hematuria, the presence of preoperative hydronephrosis, the laterality and location of the primary tumor, tumor stage, tumor grade, and the presence of angiolympathic invasion, concurrent carcinoma in situ (CIS), tumor necrosis and positive surgical margin.

The primary outcomes of this study included disease progression-free survival (PFS), overall survival (OS) and cancer-specific survival (CSS), while the secondary outcomes were urothelial recurrence-free survival (URFS). Disease progression was defined as tumor recurrence in the surgical region, regional lymph nodes, and/or distant metastases. Distant metastasis was defined as recurrent disease detected by imaging outside the bladder or the contralateral upper urinary tract. Recurrence in the bladder or the contralateral upper urinary tract was defined as urothelial recurrence. OS and CSS were defined as the time from RNU to death from any cause and specifically from UTUC, respectively. PFS and URFS were defined as the time from treatment initiation to disease progression and urothelial recurrence, respectively.

### Follow-up protocol

Patients who underwent RNU were followed up every 3 months for the first year, every 6 months from the second to fifth year, and annually after that. Follow-up examinations included history taking, physical examination, laboratory evaluation, urinary cytology, cystoscopy, and chest and abdomen/pelvic computed tomography (CT) scan based radiographic evaluations. Additional radiographic evaluations such as bone scintigraphy or MRI were performed when clinically indicated. Disease recurrence was identified based on radiography, endoscopy, or biopsy results.

### Statistical analyses

All statistical analyses were performed using IBM SPSS version 27.0 (IBM Corp., Armonk, NY, USA). The test for normality was performed with the Shapiro–Wilk test. Continuous variables were expressed as median (interquartile range). Categorical variables are presented as percentages. Continuous variables were compared among groups using the Mann–Whitney U test, while categorical variables were compared using the Chi-square test or Fisher's exact test. The Kaplan–Meier method was used to analyze the effects of CT-based features on OS, CSS, PFS and URFS with a log-rank test. Univariate and multivariate Cox regression analyses were utilized to evaluate independent predictors for overall death, cancer-specific death, progression, and urothelial recurrence. Statistical significance was set at a two-tailed *p*-value < 0.05.

## Results

The median age of all patients was 69 years, with a median follow-up duration of 46.0 months. Among 519 patients in our cohort, variant histology was present in 84 (16.2%) patients. The baseline clinical characteristics of pure UC and variant histology groups are shown in Table 1. The variant histology group showed a higher rate of hypertension (63.1% vs 50.1%, *p* = 0.029) and hydronephrosis (72.3% vs 60.0%, *p* = 0.035) preoperatively. However, other clinical features had no significant differences according to variant histology status.

**Table 1:** Comparison of baseline demographic and preoperative characteristics between the patients with pure urothelial carcinoma and those with variant histology who underwent radical nephroureterectomy for upper tract urothelial carcinoma

Characteristics	Pure UC group	Variant histology group	Overall	*p*-value
Patients, n (%)	435 (83.8%)	84 (16.2%)	519 (100.0%)	
Age, years	69 (62–75)	69 (63–77)	69 (62–75)	0.495^a^
Sex, n (%)				0.842^b^
Male	306 (70.3%)	60 (71.4%)	366 (70.5%)	
Female	129 (29.7%)	24 (28.6%)	153 (29.5%)	
BMI, kg/m^2^	24.2 (22.0–26.3)	24.0 (21.7–25.9)	24.2 (22.0–26.2)	0.535^a^
DM, n (%)	90 (20.7%)	20 (23.8%)	110 (21.2%)	0.522^b^
HTN, n (%)	218 (50.1%)	53 (63.1%)	271 (52.2%)	0.029^b^
Smoking status, n (%)				
Never smoker	365 (83.9%)	78 (92.9%)	441(85.0%)	0.080^c^
Former/current smoker	70 (16.1%)	6 (7.1%)	76(14.6%)	
History of NMIBC, n (%)	83 (19.2%)	21 (25.3%)	104 (20.2%)	0.202^b^
ECOG PS, n (%)				0.436^b^
0	31 (7.1%)	3 (3.6%)	34 (6.6%)	
1	397 (91.3%)	79 (94.0%)	476 (91.7%)	
≥ 2	7 (1.6%)	2 (2.4%)	9 (1.7%)	
Laterality of UTUC, n (%)				0.804^c^
Right	231 (53.1%)	47 (56.0%)	278 (53.6%)	
Left	202(46.4%)	37 (44.0%)	239 (46.1%)	
Bilateral	2 (0.5%)	0	2 (0.4%)	
Location of UTUC, n (%)				0.770^b^
Renal pelvis	166 (38.2%)	31 (36.9%)	197 (38.0%)	
Ureter	219 (50.3%)	41 (48.8%)	260 (50.1%)	
Both	50 (11.5%)	12 (14.3%)	62 (11.9%)	
Gross hematuria, n (%)	305 (70.1%)	57 (67.9%)	362 (69.7%)	0.680^b^
Hydronephrosis, n (%)	261 (60.0%)	60 (72.3%)	324 (62.5%)	0.035^b^

*UC* urothelial carcinoma, *BMI* body mass index, *DM* diabetes mellitus, *HTN* hypertension, *NMIBC* Non-muscle-invasive bladder cancer, *ECOG PS* Eastern Cooperative Oncology Group Performance status, *UTUC* upper tract urothelial carcinoma

^a^Mann-Whitney U test

^b^chi-square test

^c^Fisher’s exact test, Data presented are median (interquartile range) or number (%)

### Perioperative and pathological outcomes

There were no significant differences in operation time (*p* = 0.386) or operation method (*p* = 0.065) between pure UC and variant histology groups (Table 2). For pathologic outcomes, there was a tendency for variant histology group to show advanced pathologic T stage (59.5% vs 33.3%, *p* < 0.001), lymphatic node metastasis (17.9% vs 7.8%, *p* = 0.015), higher tumor grade (96.4% vs 85.7%, *p* = 0.025), and a higher rate of angiolymphatic invasion (41.7% vs 25.7%, *p* = 0.003), tumor necrosis (57.1% vs 29.0%, *p* < 0.001) and positive surgical margin (13.1% vs 5.7%, *p* = 0.015). Among a total of 84 patients with variant histology, the most frequent variant type was squamous cell differentiation in 54 patients (64.3%), followed by glandular differentiation (n = 21, 25.0%) and sarcomatoid variant (n = 2, 2.4%). All histologic variants were present mixed with UC.

**Table 2:** Comparison of perioperative characteristics and survival outcomes between the patients with pure urothelial carcinoma and those with variant histology who underwent radical nephroureterectomy for upper tract urothelial carcinoma

Characteristics	Pure UC group	Variant histology group	Overall	*p*-value
Patients, n (%)	435 (83.8%)	84 (16.2%)	519 (100.0%)	
Operation time, minutes	215 (145–260)	213 (185–265)	215 (175–264)	0.386^a^
Operation method, n (%)				0.065^b^
Open	134 (30.8%)	31 (36.9%)	165 (31.8%)	
Laparoscopic	123 (28.3%)	30 (35.7%)	153 (29.5%)	
Robotic	178 (40.9%)	23 (27.4%)	201 (38.7%)	
Tumor size, cm	3.5 (2.4–5.4)	4.0 (2.5–5.0)	3.5 (2.4–5.3)	0.836^a^
Tumor multifocality, n (%)	27 (6.2%)	3 (3.6%)	30 (5.8%)	0.450^c^
Pathologic T stage, n (%)				** < 0.001**^**a**^
≤ T2	290 (66.7%)	34 (40.5%)	324 (62.4%)	
T3-4	145 (33.3%)	50 (59.5%)	195 (37.6%)	
Pathologic N stage, n (%)				**0.015**^**a**^
pNx	311 (71.5%)	55 (65.5%)	366 (70.5%)	
pN0	90 (20.7%)	14 (16.7%)	104 (20.0%)	
pN1-2	34 (7.8%)	15 (17.9%)	49 (9.4%)	
Tumor grade, n (%)				**0.025**^**b**^
Low	58 (13.3%)	3 (3.6%)	61 (11.8%)	
High	373 (85.7%)	81 (96.4%)	454 (87.5%)	
Unknown	4 (0.9%)	0	4 (0.8%)	
Angiolymphatic invasion, n (%)	112 (25.7%)	35 (41.7%)	147 (28.3%)	**0.003**^**a**^
Concurrent CIS, n (%)	64 (14.7%)	9 (10.7%)	73 (14.1%)	0.335^a^
Tumor necrosis, n (%)	126 (29.0%)	48 (57.1%)	174 (33.5%)	** < 0.001**^**b**^
Variant histology type, n (%)				
Squamous differentiation		54 (64.3%)		
Glandular differentiation		21 (25.0%)		
Sarcomatoid variant		2 (2.4%)		
Mixed type		7 (8.3%)		
Positive surgical margin, n (%)	25 (5.7%)	11 (13.1%)	36 (6.9%)	**0.015**^**a**^
Follow-up, months	50.0 (28.0–80.0)	18.0 (9.3–56.3)	46.0 (20.0–75.0)	** < 0.001**^**a**^
Adjuvant chemotherapy, n (%)	41 (9.4%)	15 (17.9%)	56 (10.8%)	**0.023**^**a**^
Survival outcomes				
Urothelial recurrence, n (%)	177 (40.7%)	21 (25.0%)	198 (38.2%)	**0.007**^**b**^
Urothelial recurrence site, n (%)				
Bladder	172 (39.5%)	18 (21.4%)	190 (36.6%)	**0.002**^**b**^
Contralateral upper urinary tract	11 (2.5%)	3 (3.6%)	14 (2.7%)	0.589^b^
MIBC recurrence, n (%)	10 (2.1%)	1 (1.2%)	11 (2.1%)	0.518^c^
Disease progression, n (%)	131 (30.1%)	54 (63.4%)	185 (35.6%)	** < 0.001**^**b**^
Disease progression site, n (%)				
Lymph node	81 (18.6%)	42 (50.0%)	123 (23.7%)	** < 0.001**^**a**^
Lung	51 (11.7%)	21 (25.0%)	72 (13.9%)	**0.001**^**a**^
Bone	34 (7.8%)	21 (25.0%)	55 (10.6%)	** < 0.001**^**a**^
Liver	29 (6.7%	21 (25.0%)	50 (9.6%)	** < 0.001**^**a**^
Brain	5 (1.1%)	4 (4.8%)	9 (1.7%)	**0.042**^**b**^
Other	63 (14.5%)	30 (35.7%)	93 (17.9%)	** < 0.001**^**a**^
Death at last follow-up, n (%)	52 (12.0%)	17 (20.2%)	69 (13.3%)	**0.041**^a^
Cancer-specific death	31 (7.1%)	12 (14.3%)	43 (8.3%)	0.078^b^
Other causes	21 (4.8%)	5 (6.0%)	26 (5.0%)	

*UC* urothelial carcinoma, *CIS* carcinoma in situ, *MIBC* muscle-invasive bladder cancer

^a^Mann-Whitney U test

^b^chi-square test

^c^Fisher’s exact test, Data presented are median (interquartile range) or number (%)

**Fig. 1:**
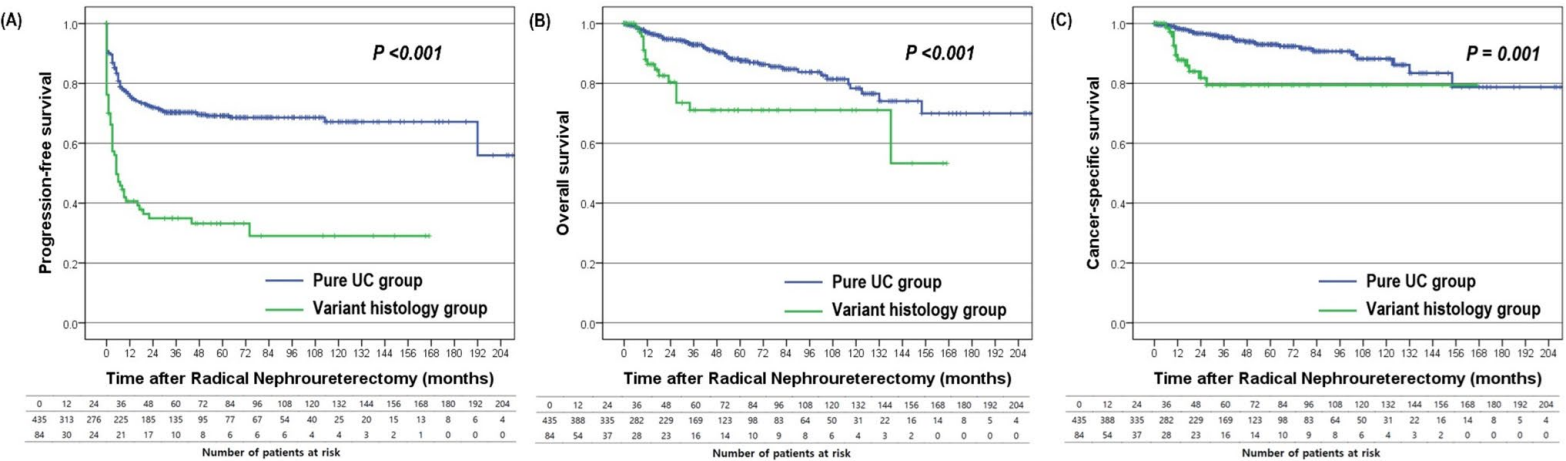
Kaplan–Meier curves comparing disease progression-free survival (**A**), overall survival (B), and cancer-specific survival (**C**) after radical nephroureterectomy for upper tract urothelial carcinoma between the pure urothelial carcinoma group and variant histology group

### Progression, cancer-specific, and overall survival outcomes

During the follow-up period, 185 patients (35.6%) experienced disease progression while 198 patients (38.2%) showed urothelial recurrence (Table 2). Urothelial recurrence occurred less frequently in patients with variant histology than in those with pure UC (25.0% vs 40.7%, *p* = 0.007). However, patients with variant histology experienced disease progression more often than those with pure UC (63.4% vs 30.1%, *p* < 0.001). The most frequent site of disease progression was lymph node (23.7%), followed by lung (13.9%), bone (10.6%), liver (9.6%) and brain (1.7%). There was no significant difference in the rate of MIBC recurrence between the two groups (*p* = 0.518).

The Kaplan-Meier survival curve analysis revealed significantly decreased PFS (log-rank test, *p* <  0.001), OS (log-rank test, *p* <  0.001) and CSS (log-rank test, *p* = 0.001) in variant histology group compared to pure UC group (Fig. 1). On multivariate Cox regression analyses, the presence of variant histology was an independent risk factor of disease progression (hazard ratio [HR] 2.23; 95% confidence interval [CI] 1.55–3.21; *p* < 0.001) (Table 3). The other predictors for disease progression included history of non-muscle-invasive bladder cancer (NMIBC) (HR 1.74; 95% CI 1.22 = 2.49; *p* = 0.002), tumor location at ureter (HR 1.47; 95% CI 1.05–2.05; *p* = 0.026), multifocality of tumor (HR 3.49; 95% CI 2.24–5.44; *p* = 0.048), pathologic T stage ≥ 3 (HR 3.61; 95% CI 2.45–5.32; *p* < 0.001), pathologic lymph node metastasis (HR 3.49; 95% CI 2.24–5.44; *p* < 0.001), angiolymphatic invasion (HR 1.54; 95% CI 1.05–2.26; *p* = 0.027), and tumor necrosis (HR 2.23; 95% CI 1.55–3.21; *p* < 0.001). Three-year PFS was 34.9% in patients with variant histology vs 70.3% in those with pure UC. For urothelial recurrence, history of NMIBC (HR 1.89; 95% CI 1.36–2.62; *p* < 0.001), tumor location at ureter (HR 1.80; 95% CI 1.30–2.49; *p* < 0.001), tumor size (HR 1.06; 95% CI 1.02–1.11; *p* = 0.004) and concurrent CIS (HR 1.56; 95% CI 1.08–2.27; *p* = 0.019) were independent risk factors (Supplementary Table 1). No significant difference was shown in URFS according to the presence of variant histology (*p* = 0.125; Supplementary Fig. 1).

**Table 3:** Univariable and multivariable Cox regression analyses of factors associated with disease progression in 519 patients who underwent radical nephroureterectomy for upper tract urothelial carcinoma

Variables	Univariable		Multivariable	
	OR (95% CI)	*p*-value	OR (95% CI)	*p*-value
Age				
< 70 years	Reference			
≥ 70 years	1.24 (0.93–1.66)	0.144		
Sex				
Female	Reference			
Male	1.10 (0.80–1.51)	0.572		
BMI	0.97 (0.93–1.01)	0.181		
DM	0.73 (0.50–1.09)	0.127		
HTN	1.22 (0.91–1.63)	0.185		
Smoking status				
Never	Reference			
Former/current	0.88 (0.58–1.34)	0.556		
History of NMIBC	1.92 (1.41–2.63)	** < 0.001**	1.74 (1.22–2.49)	**0.002**
Gross hematuria	0.60 (0.44–0.80)	**0.001**		
Hydronephrosis	1.60 (1.16–2.20)	**0.004**		
Tumor location				
Renal pelvis	Reference		Reference	
Ureter	1.46 (1.05–2.02)	**0.023**	1.47 (1.05–2.05)	**0.026**
Tumor multifocality	1.77 (1.06–2.96)	**0.029**	3.49 (2.24–5.44)	**0.048**
Tumor size	1.01 (0.96–1.05)	0.787		
pT stage				
≤ pT2	Reference		Reference	
pT3-4	5.21 (3.81–7.11)	** < 0.001**	3.61 (2.45–5.32)	** < 0.001**
pN stage				
pN0	Reference		Reference	
pN +	7.63 (5.33–10.94)	** < 0.001**	3.49 (2.24–5.44)	** < 0.001**
Tumor grade				
Low	Reference			
High	4.88 (2.16–11.02)	** < 0.001**		
Angiolymphatic invasion	4.32 (3.22–5.78)	** < 0.001**	1.54 (1.05–2.26)	**0.027**
Concurrent CIS	1.71 (1.19–2.46)	**0.003**		
Tumor necrosis	2.65 (1.98–3.55)	** < 0.001**	1.58 (1.12–2.22)	**0.010**
Positive surgical margin	2.98 1.95–4.55)	** < 0.001**		
Variant histology	3.04 (2.21–4.19)	** < 0.001**	2.23 (1.55–3.21)	** < 0.001**
Adjuvant chemotherapy	1.42 (0.95–2.11)	0.085		

*OR* odds ratio, *CI* confidence interval, *BMI* body mass index, *DM* diabetes mellitus, *HTN* hypertension, *NMIBC* Non-muscle-invasive bladder cancer, *CIS* carcinoma in situ

There were 69 deaths (13.3%) in a whole cohort during the follow-up period, with 43 (8.3%) cancer-specific deaths. Overall death occurred in 17 patients (20.2%) with variant histology vs 52 patients (12.0%) with pure UC (*p* = 0.041) (Table 2). Three-year OS and CSS were 71.1% and 79.4%, respectively, in patients with variant histology vs 92.9% and 95.4% in patients with pure UC. The presence of variant histology was also associated with worse cancer-specific survival (HR 2.67; 95% CI 1.35–5.30; *p* = 0.005) and overall survival (HR 2.22; 95% CI 1.27–3.88; *p* = 0.005) after adjusting the other clinicopathological parameters (Tables 4, 5). Besides variant histology, history of NMIBC (HR 2.06; 95% CI 1.08–3.93; *p* = 0.028), pathologic T stage ≥ 3 (HR 3.42; 95% CI 1.67–6.99; *p* = 0.001), pathologic lymph node metastasis (HR 2.49; 95% CI 1.17–5.32; *p* = 0.018) and tumor necrosis (HR 2.72; 95% CI 1.41–5.28; *p* = 0.003) were significant predictors for cancer-specific mortality while patients’ age of 70 or more (HR 2.06; 95% CI 1.25–3.39; *p* = 0.005), pathologic T stage ≥ 3 (HR 2.28; 95% CI 1.28–4.06; *p* = 0.005) and angiolymphatic invasion (HR 1.92; 95% CI 1.08–3.41; *p* = 0.027) were associated with overall mortality.

**Table 4:** Univariable and multivariable Cox regression analyses of factors associated with cancer-specific mortality in 519 patients who underwent radical nephroureterectomy for upper tract urothelial carcinoma

Variables	Univariable		Multivariable	
	OR (95% CI)	*p*-value	OR (95% CI)	*p*-value
Age				
< 70 years	Reference			
≥ 70 years	1.55 (0.84–2.85)	0.159		
Sex				
Female	Reference			
Male	0.95 (0.49–1.82)	0.868		
BMI	1.02 (0.93–1.12)	0.709		
DM	0.70 (0.29–1.65)	0.413		
HTN	1.84 (0.99–3.42)	0.053		
Smoking status				
Never	Reference			
Former/current	0.90 (0.38–2.14)	0.815		
ECOG PS				
0	Reference			
≥ 1	2.92 (0.40–21.23)	0.290		
History of NMIBC	2.32 (1.26–4.29)	**0.007**	2.06 (1.08–3.93)	**0.028**
Gross hematuria	0.63 (0.34–1.16)	0.138		
Hydronephrosis	1.38 (0.72–2.64)	0.336		
Tumor location				
Renal pelvis	Reference			
Ureter	0.85 (0.44–1.65)	0.627		
Tumor multifocality	1.03 (0.32–3.34)	0.961		
Tumor size	1.06 (1.00–1.13)	0.061		
pT stage				
≤ pT2	Reference		Reference	
pT3-4	5.67 (2.95–10.90)	** < 0.001**	3.42 (1.67–6.99)	**0.001**
pN stage				
pN0	Reference			
pN +	7.98 (4.09–15.60)	** < 0.001**	2.49 (1.17–5.32)	**0.018**
Tumor grade				
Low	Reference			
High	8.25 (1.13–60.24)	**0.037**		
Angiolymphatic invasion	5.05 (2.75–9.28)	** < 0.001**		
Concurrent CIS	1.01 (0.42–2.39)	0.988		
Tumor necrosis	4.39 (2.35–8.18)	** < 0.001**	2.72 (1.41–5.28)	**0.003**
Positive surgical margin	3.79 (1.59–9.04)	**0.003**		
Variant histology	3.05 (1.56–5.95)	**0.001**	2.67 (1.35–5.30)	**0.005**
Adjuvant chemotherapy	0.78 (0.30–2.00)	0.600		

*OR* odds ratio, *CI* confidence interval, *BMI* body mass index, *DM* diabetes mellitus, *HTN* hypertension, *ECOG PS* Eastern Cooperative Oncology Group Performance status, *NMIBC* Non-muscle-invasive bladder cancer, *CIS* carcinoma in situ

**Table 5:** Univariable and multivariable Cox regression analyses of factors associated with overall mortality in 519 patients who underwent radical nephroureterectomy for upper tract urothelial carcinoma

Variables	Univariable		Multivariable	
	OR (95% CI)	*p*-value	OR (95% CI)	*p*-value
Age				
< 70 years	Reference		Reference	
≥ 70 years	2.49 (1.53–4.07)	** < 0.001**	2.06 (1.25–3.39)	**0.005**
Sex				
Female	Reference			
Male	1.16 (0.68–1.99)	0.586		
BMI	0.92 (0.85–0.99)	**0.032**		
DM	0.65 (0.32–1.32)	0.236		
HTN	1.52 (0.94–2.46)	0.084		
Smoking status				
Never	Reference			
Former/current	0.72 (0.35–1.51)	0.390		
ECOG PS				
0	Reference			
≥ 1	4.60 (0.64–33.16)	0.130		
History of NMIBC	1.55 (0.93–2.60)	0.092		
Gross hematuria	0.70 (0.42–1.14)	0.151		
Hydronephrosis	1.55 (0.91–2.63)	0.104		
Tumor location				
Renal pelvis	Reference			
Ureter	0.70 (0.42–1.17)	0.172		
Tumor multifocality	0.84 (0.31–2.31)	0.737		
Tumor size	1.04 (0.99–1.10)	0.152		
pT stage				
≤ pT2	Reference		Reference	
pT3-4	3.90 (2.40–6.35)	** < 0.001**	2.28 (1.28–4.06)	**0.005**
pN stage				
pN0	Reference		Reference	
pN +	5.31 (2.96–9.54)	** < 0.001**	1.86 (0.95–3.65)	0.069
Tumor grade				
Low	Reference			
High	2.54 (1.02–6.36)	**0.046**		
Angiolymphatic invasion	4.00 (2.49–6.43)	** < 0.001**	1.92 (1.08–3.41)	**0.027**
Concurrent CIS	0.80 (0.38–1.67)	0.553		
Tumor necrosis	2.60 (1.60–4.23)	** < 0.001**	1.66 (0.99–2.80)	0.056
Positive surgical margin	2.71 (1.24–5.96)	**0.013**		
Variant histology	2.59 (1.49–4.48)	**0.001**	2.22 (1.27–3.88)	**0.005**
Adjuvant chemotherapy	0.77 (0.36–1.62)	0.485		

*OR* odds ratio, *CI* confidence interval, *BMI* body mass index, *DM* diabetes mellitus, *HTN* hypertension, *ECOG PS* Eastern Cooperative Oncology Group Performance status, *NMIBC* Non-muscle-invasive bladder cancer, *CIS* carcinoma in situ

To compare the efficacy of adjuvant chemotherapy in patients with variant histology, we initially compared baseline clinicopathologic features according to the performance of adjuvant chemotherapy, which showed no significant differences in general (all *p* > 0.05; Supplementary Table 2). There were no statistically significant differences in PFS (*p* = 0.775), URFS (*p* = 0.433), OS (*p* = 0.724) and CSS (*p* = 0.626) according to adjuvant chemotherapy among patients with variant histology (Supplementary Fig. 2).

## Discussion

In the present study, 519 patients who underwent RNU for UTUC were enrolled to evaluate retrospectively the impact of variant histology on survival outcomes. Our result showed that the prevalence of variant histology was 16.2% among the patients who underwent RNU, similar to previous studies with a wide range from 7.5 to 40% [8, 9, 15–17]. The most frequent subtype of variant histology was squamous differentiation, followed by glandular differentiation and other variants congruent with results of previous studies [17].

In general, variant histology has been acknowledged to be associated with adverse features. According to our results, patients with variant histology showed significantly higher rates of hydronephroses preoperatively, which has been reported to be related to the aggressiveness of UTUC [18]. For pathologic outcomes, patients with variant histology presented advanced T stage, higher tumor grade, and higher rates of lymph node metastasis, angiolymphatic invasion, tumor necrosis, and positive surgical margin, which have been well-recognized as adverse pathologic features [5]. The previous meta-analysis study supports our data, showing that UC with variant histology is usually associated with advanced-stage necrosis and angiolymphatic invasion [2]. These results suggest that variant histology might be related to other adverse pathologic features.

Recently, several studies have reported the effect of variant histology on prognostic outcomes in patients who underwent RNU for UTUC. However, the results of previous studies were somewhat controversial. Furthermore, several studies [8–10] advocated that variant histology was correlated with poor prognosis, while others [10, 11] failed to show a significant association. Nogueira et al. reported that UTUC with variant histology was associated with advanced stage and poor survival. However, after adjusting for the pathologic T stage, variant histology was not significantly associated with CSS [16].

In our study, patients with variant histology were likely to show higher rate of disease progression, cancer-specific and overall mortality, but not urothelial recurrence and MIBC recurrence. After adjusting the other adverse pathologic features on multivariate Cox regression analyses, variant histology was identified as an independent risk factor for disease progression, cancer-specific mortality, and overall mortality after RNU, while was not associated with urothelial recurrence. Our results were consistent with those of the previous meta-analyses [2]. Therefore, it would be reasonable to consider additional therapeutic options for patients with variant histology after RNU.

There are several studies on adjuvant chemotherapy in UTUC patients with variant histology. However, those studies provided conflicting results. Chung et al. showed that the significant differences in OS, CSS, and recurrence-free survival between patients with pure UC vs variant histology disappeared after adjuvant chemotherapy, concluding that adjuvant chemotherapy improved survival outcomes in patients with UTUC with variant histology [9]. Lo et al. [19] also reported that adjuvant chemotherapy provided significant survival benefits in CSS and disease-free survival for patients diagnosed with UTUC with variant histology. On the contrary, Rink and colleagues reported no significant differences in survival outcomes between pure UC and variant histology groups, despite variant histology correlated with adverse features [20]. Kim et al. [8] also demonstrated that patients with variant histology still showed worse survival outcomes than those with pure UC after adjusting for other confounding features. In our cohort, there were generally no significant differences in baseline clinicopathologic features according to adjuvant chemotherapy status among patients with variant histology. However, adjuvant chemotherapy failed to show significant benefits in PFS, URFS, OS, and CSS among patients with variant histology. Our results suggest that novel adjuvant treatments other than chemotherapy might have to be considered for patients with variant histology. These results should be verified with a longer follow-up period and a larger population volume.

Our study had limitations, mainly due to the nature of the retrospective design. Considering that the present study was single institution based, the radiologic and pathologic evaluation protocol and surgical technique could be homogenous; however, the risk of selection bias could not be excluded. In addition, the comparison among subgroups of variant histology was not available due to the small number of cases. Further study involving a larger population is required.

Moreover, we could not assess the efficacy of the other adjuvant therapy besides adjuvant chemotherapy, such as radiotherapy or novel immunotherapy. Furthermore, the current study also could not include the results of ureteroscopic biopsy before RNU because most patients omitted preoperative ureteroscopic evaluation or biopsy. Therefore, we could not evaluate the diagnostic accuracy of ureteroscopic biopsy for variant histology, although it might be inferior in comparison with definitive pathologic results after RNU.

## Conclusion

In our cohort, variant histology accounted for 16.2% of all UTUC patients who underwent RNU. Variant histology was associated with adverse pathologic features and poorer survival outcomes, such as overall survival, cancer-specific survival, and progression-free survival after adjusting other clinicopathologic factors. Adjuvant chemotherapy after RNU failed to provide significant survival gains for UTUC patients with variant histology. These results suggest that UTUC patients with variant histology may be recommended a close follow-up schedule and adjuvant therapy other than chemotherapy after RNU.

## Supplementary Information

Below is the link to the electronic supplementary material.

Supplementary Supplementary Figure 1. Kaplan-Meier curves comparing urothelial recurrence-free survival after radical nephroureterectomy for upper tract urothelial carcinoma between the pure urothelial carcinoma group and variant histology group file1 (JPG 83 KB)

Supplementary Supplementary Figure 2. Kaplan-Meier curves comparing progression-free survival (A), urothelial recurrence-free survival (B), overall survival (C), and cancer-specific survival (D) after radical nephroureterectomy for upper tract urothelial carcinoma according to adjuvant chemotherapy in patients with variant histology file2 (JPG 309 KB)

Supplementary file3 (DOCX 29 KB)

Supplementary file4 (DOCX 28 KB)

## Data Availability

The datasets analysed during the current study are available from the corresponding author.

## Abbreviations

UTUC: Upper urinary tract urothelial carcinoma
RNU: Radical nephroureterectomy
MIBC: Muscle-invasive bladder cancer
NMIBC: Non-muscle-invasive bladder cancer
PFS: Progression-free survival
URFS: Urothelial recurrence-free survival
CSS: Cancer-specific survival
OS: Overall survival

## References

[1] Petros FG (2020) Epidemiology, clinical presentation, and evaluation of upper-tract urothelial carcinoma. Transl Androl Urol 9:1794–1798. 10.21037/tau.2019.11.22 32944542 10.21037/tau.2019.11.22 PMC7475674

[2] Mori K, Janisch F, Parizi MK et al (2020) Prognostic value of variant histology in upper tract urothelial carcinoma treated with nephroureterectomy: a systematic review and meta-analysis. J Urol 203:1075–1084. 10.1097/JU.0000000000000523 31479406 10.1097/JU.0000000000000523

[3] Rosiello G, Palumbo C, Knipper S et al (2020) Contemporary conditional cancer-specific survival after radical nephroureterectomy in patients with nonmetastatic urothelial carcinoma of upper urinary tract. J Surg Oncol 121:1154–1161. 10.1002/jso.25877 32107785 10.1002/jso.25877

[4] Li X, Cui M, Gu X et al (2020) Pattern and risk factors of local recurrence after nephroureterectomy for upper tract urothelial carcinoma. World J Surg Oncol 18:114. 10.1186/s12957-020-01877-w 32473636 10.1186/s12957-020-01877-w PMC7261378

[5] Rouprêt M, Babjuk M, Burger M et al (2021) European association of urology guidelines on upper urinary tract urothelial carcinoma: 2020 update. Eur Urol 79:62–79. 10.1016/j.eururo.2020.05.042 32593530 10.1016/j.eururo.2020.05.042

[6] Black AJ, Black PC (2020) Variant histology in bladder cancer: diagnostic and clinical implications. Transl Cancer Res 9:6565–6575. 10.21037/tcr-20-2169 35117266 10.21037/tcr-20-2169 PMC8798576

[7] Green DA, Rink M, Xylinas E et al (2013) Urothelial carcinoma of the bladder and the upper tract: disparate twins. J Urol 189:1214–1221. 10.1016/j.juro.2012.05.079 23023150 10.1016/j.juro.2012.05.079

[8] Kim JK, Moon KC, Jeong CW et al (2017) Variant histology as a significant predictor of survival after radical nephroureterectomy in patients with upper urinary tract urothelial carcinoma. Urol Oncol 35:458.e9-458.e15. 10.1016/j.urolonc.2017.02.010 28347659 10.1016/j.urolonc.2017.02.010

[9] Chung HS, Hwang EC, Kim MS et al (2019) Effects of variant histology on the oncologic outcomes of patients with upper urinary tract carcinoma after radical nephroureterectomy: a propensity score-matched analysis. Clin Genitourin Cancer 17:e394–e407. 10.1016/j.clgc.2018.11.015 30782419 10.1016/j.clgc.2018.11.015

[10] Takemoto K, Hayashi T, Hsi RS et al (2022) Histological variants and lymphovascular invasion in upper tract urothelial carcinoma can stratify prognosis after radical nephroureterectomy. Urol Oncol 40:539.e9-539.e16. 10.1016/j.urolonc.2022.08.010 36244916 10.1016/j.urolonc.2022.08.010

[11] Sakano S, Matsuyama H, Kamiryo Y et al (2015) (2015) Impact of variant histology on disease aggressiveness and outcome after nephroureterectomy in Japanese patients with upper tract urothelial carcinoma. Int J Clin Oncol 20:362–368. 10.1007/s10147-014-0721-3 24964974 10.1007/s10147-014-0721-3

[12] Tang Q, Xiong G, Li X et al (2016) The prognostic impact of squamous and glandular differentiation for upper tract urothelial carcinoma patients after radical nephroureterectomy. World J Urol 34:871–877. 10.1007/s00345-015-1715-0 26497969 10.1007/s00345-015-1715-0

[13] Song SH, Ye CH, Lee S et al (2019) Association between lymphovascular invasion and oncologic outcomes among upper urinary tract urothelial carcinoma patients who underwent radical nephroureterectomy. J Cancer Res Clin Oncol 145:2863–2870. 10.1007/s00432-019-03020-z 31501983 10.1007/s00432-019-03020-z PMC11810388

[14] Humphrey PA, Moch H, Cubilla AL et al (2016) The 2016 WHO classification of tumours of the urinary system and male genital organs-part B: prostate and bladder tumours. Eur Urol 70:106–119. 10.1016/j.eururo.2016.02.028 26996659 10.1016/j.eururo.2016.02.028

[15] Zamboni S, Foerster B, Abufaraj M et al (2019) (2019) Incidence and survival outcomes in patients with upper urinary tract urothelial carcinoma diagnosed with variant histology and treated with nephroureterectomy. BJU Int 124:738–745. 10.1111/bju.14751 30908835 10.1111/bju.14751

[16] Nogueira LM, Yip W, Assel MJ et al (2022) Survival impact of variant histology diagnosis in upper tract urothelial carcinoma. J Urol 208:813–820. 10.1097/JU.0000000000002799 35686817 10.1097/JU.0000000000002799 PMC10163931

[17] Rolim I, Henriques V, Rolim N et al (2020) Clinicopathologic analysis of upper urinary tract carcinoma with variant histology. Virchows Arch 477:111–120. 10.1007/s00428-020-02745-4 31950242 10.1007/s00428-020-02745-4

[18] Soria F, Shariat SF, Lerner SP et al (2017) 2017) Epidemiology, diagnosis, preoperative evaluation and prognostic assessment of upper-tract urothelial carcinoma (UTUC. World J Urol 35:379–387. 10.1007/s00345-016-1928-x 27604375 10.1007/s00345-016-1928-x

[19] Lo CW, Li WM, Ke HL et al (2022) Impact of adjuvant chemotherapy on variant histology of upper tract urothelial carcinoma: a propensity score-matched cohort analysis. Front Oncol 12:843715. 10.3389/fonc.2022.843715 35530335 10.3389/fonc.2022.843715 PMC9072967

[20] Rink M, Robinson BD, Green DA et al (2012) Impact of histological variants on clinical outcomes of patients with upper urinary tract urothelial carcinoma. J Urol 188:398–404. 10.1016/j.juro.2012.04.009 22698626 10.1016/j.juro.2012.04.009

