# Supplementary figures and images for: Evaluation of histological variants of upper tract urothelial carcinoma as prognostic factor after radical nephroureterectomy
Source: World J Urol. 2024 Apr 9;42(1):225. doi: 10.1007/s00345-024-04878-6 (PMC11003889; doi:10.1007/s00345-024-04878-6)

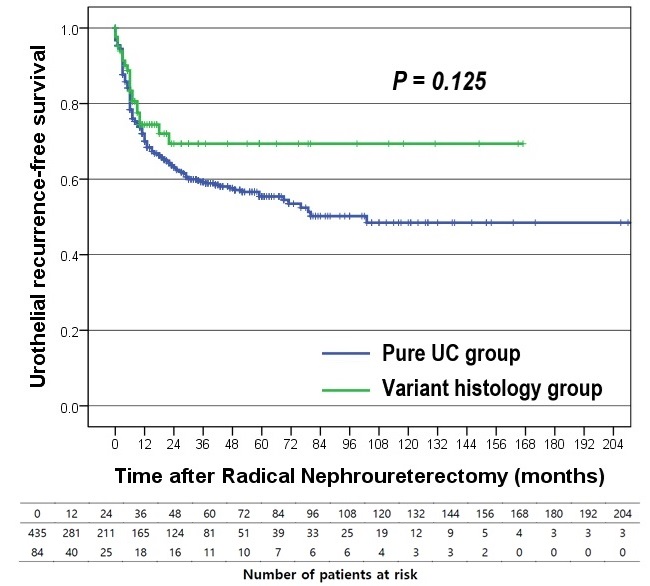

Supplement: Supplementary file 1 — Supplementary Supplementary Figure 1. Kaplan-Meier curves comparing urothelial recurrence-free survival after radical nephroureterectomy for upper tract urothelial carcinoma between the pure urothelial carcinoma group and variant histology group file1 (JPG 83 KB) [file 345_2024_4878_MOESM1_ESM.jpg]

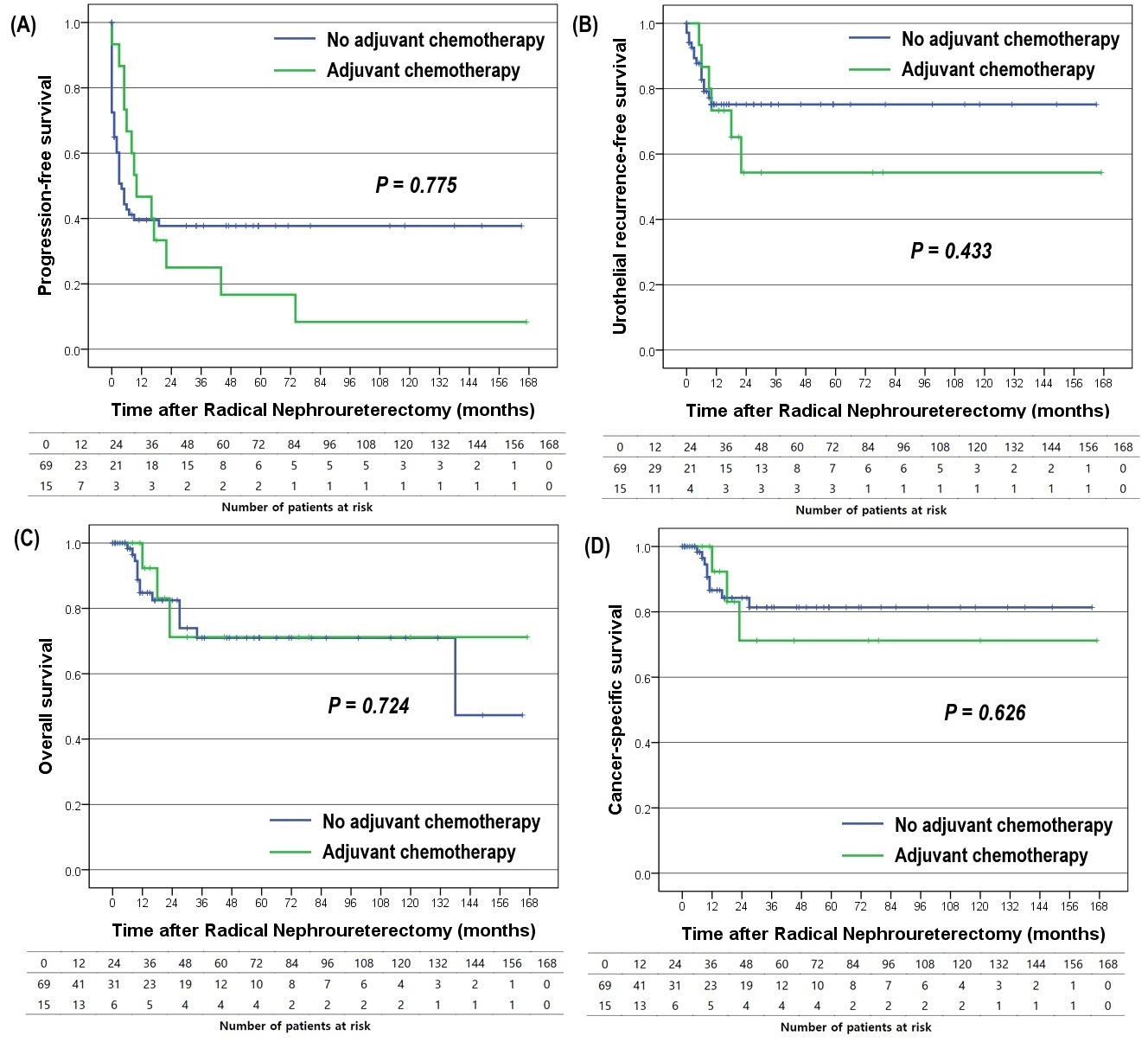

Supplement: Supplementary file 2 — Supplementary Supplementary Figure 2. Kaplan-Meier curves comparing progression-free survival (A), urothelial recurrence-free survival (B), overall survival (C), and cancer-specific survival (D) after radical nephroureterectomy for upper tract urothelial carcinoma according to adjuvant chemotherapy in patients with variant histology file2 (JPG 309 KB) [file 345_2024_4878_MOESM2_ESM.jpg]
