# Supplementary material for: Evaluation of histological variants of upper tract urothelial carcinoma as prognostic factor after radical nephroureterectomy
Source: World J Urol. 2024 Apr 9;42(1):225. doi: 10.1007/s00345-024-04878-6 (PMC11003889; doi:10.1007/s00345-024-04878-6)
Supplement: Supplementary file 3 — Supplementary file3 (DOCX 29 KB) [file 345_2024_4878_MOESM3_ESM.docx]

**Supplementary Table 1 Univariable and multivariable Cox regression analyses of factors associated with urothelial recurrence-free survival of 519 patients who underwent radical nephroureterectomy.**

| **Variables** | **Univariable** | | **Multivariable** | |
| --- | --- | --- | --- | --- |
|  | **OR (95% CI)** | ***p*-value** | **OR (95% CI)** | ***p*-value** |
| **Age** |  |  |  |  |
| **<70 years** | Reference |  |  |  |
| **≥70 years** | 0.94 (0.71-1.24) | 0.647 |  |  |
| **Sex** |  |  |  |  |
| **Female** | Reference |  |  |  |
| **Male** | 1.36 (0.99-1.88) | 0.062 |  |  |
| **BMI** | 0.97 (0.93-1.02) | 0.191 |  |  |
| **DM** | 1.10 (0.78-1.54) | 0.599 |  |  |
| **HTN** | 1.24 (0.94-1.64) | 0132 |  |  |
| **Smoking status** |  |  |  |  |
| **Never** | Reference |  |  |  |
| **Former/current** | 1.16 (0.80-1.69) | 0.429 |  |  |
| **History of NMIBC** | 2.38 (1.77-3.21) | **<0.001** | 1.89 (1.36-2.62) | **<0.001** |
| **Gross hematuria** | 1.10 (0.81-1.50) | 0.549 |  |  |
| **Hydronephrosis** | 1.45 (1.07-1.96) | **0.015** |  |  |
| **Tumor location** |  |  |  |  |
| **Renal pelvis** | Reference |  | Reference |  |
| **Ureter** | 1.93 (1.40-2.66) | **<0.001** | 1.80 (1.30-2.49) | **<0.001** |
| **Tumor multifocality** | 2.13 (1.31-3.45) | **0.002** |  |  |
| **Tumor size** | 1.08 (1.04-1.12) | **<0.001** | 1.06 (1.02-1.11) | **0.004** |
| **pT stage** |  |  |  |  |
| **≤pT2** | Reference |  |  |  |
| **pT3-4** | 0.90 (0.66-1.22) | 0.484 |  |  |
| **pN stage (yes vs. no)** |  |  |  |  |
| **pN0** | Reference |  |  |  |
| **pN+** | 0.82 (0.46-1.46) | 0.495 |  |  |
| **Tumor grade (High vs. Low)** |  |  |  |  |
| **Low** | Reference |  |  |  |
| **High** | 1.01 (0.67-1.53) | 0.946 |  |  |
| **Angiolymphatic invasion** | 0.91 (0.65-1.27) | 0.584 |  |  |
| **Concurrent CIS** | 1.82 (1.27-2.58) | **0.001** | 1.56 (1.08-2.27) | **0.019** |
| **Tumor necrosis** | 0.76 (0.55-1.05) | 0.095 |  |  |
| **Positive surgical margin** | 1.40 (0.80-2.46) | 0.240 |  |  |
| **Variant histology** | 0.71 (0.45-1.11) | 0.133 |  |  |
| **Adjuvant chemotherapy** | 1.15 (0.77-1.73) | 0.490 |  |  |

OR: odds ratio, CI: confidence interval, BMI: body mass index, DM: diabetes mellitus, HTN: hypertension, NMIBC: Non-muscle-invasive bladder cancer, CIS: carcinoma in situ.
