# Supplementary material for: Evaluation of histological variants of upper tract urothelial carcinoma as prognostic factor after radical nephroureterectomy
Source: World J Urol. 2024 Apr 9;42(1):225. doi: 10.1007/s00345-024-04878-6 (PMC11003889; doi:10.1007/s00345-024-04878-6)
Supplement: Supplementary file 4 — Supplementary file4 (DOCX 28 KB) [file 345_2024_4878_MOESM4_ESM.docx]

**Supplementary table 2. Comparison of baseline clinicopathologic characteristics of the patients with variant histology who underwent radical nephroureterectomy for upper tract urothelial carcinoma according with adjuvant chemotherapy**

| **Characteristics** | **Without Adjuvant chemotherapy** | **Adjuvant chemotherapy** | **Overall** | ***p*-value** |
| --- | --- | --- | --- | --- |
| **Patients, n (%)** | 69 (82.1%) | 15 (17.9%) | 84 (100.0%) | 0.069^a^ |
| **Age, years** | 70.0 (63.0-79.5) | 68.0 (55.5-69.5) | 69.0 (63.0-77.0) |  |
| **Sex, n (%)** |  |  |  | 0.212^c^ |
| **Male** | 47 (68.1%) | 13 (86.7%) | 60 (71.4%) |  |
| **Female** | 22 (31.9%) | 2 (13.3%) | 24 (28.6%) |  |
| **BMI, kg/m^2^** |  |  |  |  |
| **DM, n (%)** | 15 (21.7%) | 5 (33.3%) | 20 (23.8%) | 0.335^c^ |
| **HTN, n (%)** | 44 (63.8%) | 9 (60.0%) | 53 (63.1%) | 0.784^b^ |
| **Smoking status, n (%)** |  |  |  | 0.290^c^ |
| **Never smoker** | 65 (94.2%) | 13 (86.7%) | 78 (92.9%) |  |
| **Former/Current smoker** | 4 (5.8%) | 2 (13.3%) | 6 (7.1%) |  |
| **History of NMIBC, n (%)** | 9 (13.0%) | 4 (26.7%) | 13 (15.5%) | 0.235^c^ |
| **ECOG PS, n (%)** |  |  |  | 0.561^c^ |
| **0** | 3 (4.3%) | 0 (0.0%) | 3 (3.6%) |  |
| **1** | 64 (92.8%) | 15 (100.0%) | 79 (94.0%) |  |
| **≥2** | 2 (2.9%) | 0 (0.0%) | 2 (2.4%) |  |
| **Laterality of UTUC, n (%)** |  |  |  | 0.424^b^ |
| **Right** | 40 (58.0%) | 7 (46.7%) | 47 (56.0%) |  |
| **Left** | 29 (42.0%) | 8 (53.3%) | 37 (44.0%) |  |
| **Location of UTUC, n (%)** |  |  |  | 0.287^b^ |
| **Renal pelvis** | 27 (39.1%) | 4 (25.7%) | 31 (36.9% |  |
| **Ureter** | 34 (49.3%) | 7 (46.7%) | 41 (48.8%) |  |
| **Both** | 8 (11.6%) | 4 (26.7%) | 12 (14.3%) |  |
| **Gross hematuria, n (%)** | 45 (65.2%) | 12 (80.0%) | 57 (67.9%) | 0.366^c^ |
| **Hydronephrosis, n (%)** | 49 (72.1%) | 11 (73.3%) | 60 (72.3%) | 1.000^c^ |
| **Tumor Size, cm** | 4.0 (2.5-6.6) | 4.0 (2.3-7.0) | 4.0 (2.5-5.0) | 0.528^a^ |
| **Tumor multifocality, n (%)** | 3 (4.3%) | 0 (0.0%) | 3 (3.6%) | 1.000^c^ |
| **Pathologic T stage, n (%)** |  |  |  | 0.534^b^ |
| **≤T2** | 29 (42.0%) | 5 (33.0%) | 34 (40.5%) |  |
| **T3-4** | 40 (58.0%) | 10 (66.7%) | 50 (59.5%) |  |
| **Pathologic N stage, n (%)** |  |  |  | 0.518^b^ |
| **pNx** | 44 (63.8%) | 11 (73.3%) | 55 (65.5%) |  |
| **pN0** | 13 (18.8%) | 1 (6.7% | 14 (16.7%) |  |
| **pN1-2** | 12 (17.4%) | 3 (20.0%) | 15 (17.9%) |  |
| **Tumor grade, n (%)** |  |  |  | 1.000^c^ |
| **Low** | 3 (4.3%) | 0 (0.0%) | 3 (3.6%) |  |
| **High** | 66 (95.7%) | 15 (100.0%) | 81 (96.4%) |  |
| **Angiolymphatic invasion, n (%)** | 28 (40.6%) | 7 (46.7%) | 35 (41.7%) | 0.665^b^ |
| **Concurrent CIS, n (%)** | 9 (13.0%) | 0 (0.0%) | 9 (10.7%) | 0.352^c^ |
| **Tumor necrosis, n (%)** | 41 (59.4%) | 7 (46.7%) | 48 (57.1%) | 0.366^b^ |
| **Positive surgical margin, n (%)** | 10 (14.5%) | 1 (6.7%) | 11 (13.1%) | 0.680^c^ |

BMI: body mass index, DM: diabetes mellitus, HTN: hypertension, ECOG PS: Eastern Cooperative Oncology Group Performance status, a: Mann-Whitney U test, b: chi-square test, c: Fisher's exact test, Data presented are median (interquartile range) or number (%)
